# Supplementary material for: A balance of positive and negative regulators determines the pace of the segmentation clock
Source: eLife. 2015 Sep 10;4:e05842. doi: 10.7554/eLife.05842 (PMC4601006; doi:10.7554/eLife.05842)
Supplement: Supplementary file 1. — This file contains further analysis of the mathematical model and notes on parameter identification. DOI: http://dx.doi.org/10.7554/eLife.05842.019 [file elife05842s001.pdf]

1 Model Development

We consider analysis of equations (1)–(3) presented in the Main Text. To recap, we define two dependent variables, $p(t)$ and $n(t)$, that are functions of time, t, and represent numbers of molecules of cytoplasmic clock protein and NICD, respectively. Although we do not explicitly describe the transcription of clock genes, we impose known transcriptional logic of clock gene expression in the mouse PSM to define the production rate of clock protein, i.e.

$$f(n,p) = \frac{\frac{n}{K_n}}{1 + \left(\frac{p}{K_p}\right)^2}. \quad (1)$$

This functional form captures the principles that NICD is necessary for transcription and clock protein represses transcription. We assume that the logic of transcriptional activation is such that the balance between clock protein and NICD sets the overall transcription rate.

The governing equations are given by

$$\frac{dp}{dt} = k_1 f(n(t-T_1), p(t-T_1)) - k_2 p(t), \quad (2)$$

$$\frac{dn}{dt} = k_3 f(n(t-T_2), p(t-T_2)) - k_4 n(t), \quad (3)$$

and all rate constants are assumed to be positive. The delay T_1 represents the time taken for changes in cytoplasmic protein number to affect the next round of protein synthesis (delays arising as a result of transcription, splicing and transport). The difference between T_1 and T_2 represents the additional time that elapses between the emergence of clock protein in the cytoplasm and the release of NICD from the cell membrane.

We rescale the dependent variables such that

$$\hat{p} = \frac{p}{K_p} \quad \hat{n} = \frac{n}{K_n}, \quad (4)$$

and, defining the parameters

$$\gamma_1 = \frac{k_1}{K_p} \quad \gamma_2 = \frac{k_3}{K_n}, \quad (5)$$

the governing equations are given by

$$\frac{d\hat{p}}{dt} = \gamma_1 \hat{f}(\hat{n}(t-T_1), \hat{p}(t-T_1)) - k_2 \hat{p}(t), \quad (6)$$

$$\frac{d\hat{n}}{dt} = \gamma_2 \hat{f}(\hat{n}(t-T_2), \hat{p}(t-T_2)) - k_4 \hat{n}(t), \quad (7)$$

where

$$\hat{f}(\hat{n}, \hat{p}) = \frac{\hat{n}}{1 + \hat{p}^2}. \quad (8)$$

For convenience, we now drop the hatted notation.

We seek the steady-states of equations (6)–(7) are find that they are given by the expressions

$$p_{ss} = 0, \quad n_{ss} = 0, \tag{9}$$

and

$$p_{ss} = \sqrt{\frac{\gamma_2}{k_4} - 1}, \quad n_{ss} = \frac{\gamma_2 k_2}{\gamma_1 k_4}\sqrt{\frac{\gamma_2}{k_4} - 1}. \tag{10}$$

Hence if

$$\gamma_2 < k_4, \tag{11}$$

the trivial steady state is the only real steady state and it can be shown that it is linearly stable. If

$$\gamma_2 > k_4, \tag{12}$$

the trivial steady state becomes unstable and the non-trivial steady-state becomes real.

2 Model parameters

In order to determine model parameters we have used estimates from previous works and fitted parameters so that the model yields experimentally reasonable levels of molecules.

Given the observations of NICD decay presented in the Main Text we set the NICD half life to be of approximately 12 minutes. It has previously been assumed that the half life of clock repressor Hes7 is 20 minutes [2]. As we could not measure the clock repressor half-life, here we assume a value similar to that of NICD.

Note that in the rescaled model equations the constants K_P and K_N are absorbed into the production constants γ_1 and γ_2, respectively, hence do not qualitatively affect model behaviour. In previous models of the somitogenesis clock ([e.g. 3]) the number of mRNA molecules is explicitly defined and two delays are defined to represent processes such as transcription, splicing and export of mRNA to the cytoplasm and the translation of mRNA into the protein. As the goal of this model is to study the effect of protein half lives on the clock period, here we do not explicitly account for mRNA but the delays are incorporated in the parameter T_1. We choose a value that is consistent with previous model [2].

For the second delay parameter T_2 we assume that the release of NICD into the cytoplasm is downstream of protein, hence $T_2 > T_1$. Based upon observations of

the relative positions of NICD and other clock components (data not shown) we approximate that this additional delay is 23 minutes. We choose the production rates k_1 and k_3 such that there are of the order of hundreds of molecules of activator and inhibitors.

Parameter	Value	Unit
k_1	123	min^{-1}
k_2	0.058	min^{-1}
k_3	134	min^{-1}
k_4	0.063	min^{-1}
K_P	125.0	Nondimensional
K_N	125.0	Nondimensional
τ_1	44.0	min
τ_2	67.0	min

Table 1: Parameter values used to solve equations (2)–(3).

3 Numerical solution

Numerical solutions of equations (2)–(3) were calculated using Matlab's delay differential equation solver 'dde23'. In order to numerically calculate the oscillator period, initial histories $\{[p(t), n(t)] = [0, 1]; -T_2 < t < 0\}$ were applied and the numerical solution were computed until oscillations reached dynamic equilibrium. Hopf bifurcation were identified using the freely available package DDEBif [1].

References

[1] Koen Engelborghs, Tatyana Luzyanina, and Giovanni Samaey. DDE-BIFTOOL v. 2.00: a Matlab package for bifurcation analysis of delay differential equations. *TW Reports*, page 61, 2001.

[2] Yukiko Harima, Yoshiki Takashima, Yuriko Ueda, Toshiyuki Ohtsuka, and Ryoichiro Kageyama. Accelerating the tempo of the segmentation clock by reducing the number of introns in the¡ i¿ hes7¡/i¿ gene. *Cell reports*, 3(1):1–7, 2013.

[3] Julian Lewis. Autoinhibition with transcriptional delay: a simple mechanism for the zebrafish somitogenesis oscillator. *Current Biology*, 13(16):1398–1408, 2003.
